# Pull-Through Behavior of Novel Additively Manufactured Sandwich Composite Inserts

**DOI:** 10.3390/ma17081884

**Published:** 2024-04-19

**Authors:** Patrick Severson, Anna Lutz, Rani Elhajjar

**Affiliations:** College of Engineering and Applied Science, University of Wisconsin-Milwaukee, 3200 N Cramer St., Milwaukee, WI 53211, USA; severs39@uwm.edu (P.S.); annalutz@uwm.edu (A.L.)

**Keywords:** sandwich composites, carbon fiber reinforced plastic, aluminum honeycomb, bolted joint

## Abstract

Joining structural components with mechanical fasteners is common in many engineering applications across all industries. This study investigates combining additive manufactured inserts with sandwich composites consisting of aluminum honeycomb cores with carbon fiber reinforced facesheets. The combination of these components offers an integrated, lightweight solution when mechanically fastening sandwich composite components using bolted joints. The experimental and numerical investigation explores the influence insert geometry has on the structural response of a sandwich composite under pull-through load scenarios. Various failure modes are observed during experimental analysis with facesheet debonding being the initial failure mode. In addition, finite element models investigate the stress fields in the honeycomb core and overall panel deflections, validating the mechanics observed experimentally. When comparing additively manufactured inserts to standard inserts, additively manufactured inserts have increases in stiffness, maximum force, and total energy absorption of 7.1%, 53.0%, and 62.3%, respectively. These results illustrate the potential of an integrated approach to mechanical joint technology by combining additively manufactured inserts with sandwich composite components using aluminum honeycomb cores.

## 1. Introduction

Sandwich composites offer a lightweight material system with high specific strength and stiffness for many engineering applications across multiple industries. These industries include automotive, aerospace, energy, marine, biomedical, and recreational. One of the most common methods of transferring loads between two or more components is using mechanical fasteners. Mechanical connections are often areas of premature failure and, therefore, require reliable fastening techniques to accommodate local bending and stress concentrations in the sandwich panel. The most common mechanical fastening technique makes use of metallic inserts, which can come in a variety of forms, and are bonded in the panel in a post-fabrication process [1]. The failure of sandwich panels with embedded inserts usually occurs due to delamination, shear rupture, or bending of the facesheets [2].

Since sandwich panels with embedded inserts generally fail due to local stress concentrations, there have been several analytical models developed to quantify these stresses. Several authors present models that use the “antiplane” sandwich plate theory that excludes the core’s transverse stiffness [3,4,5,6]. However, for problems that include inserts, through-thickness stiffness is important and should not be neglected. Several “high-order” approaches have been proposed to include the transverse stiffness, where the core is modeled as a transversely isotropic solid for both beams and plates [7,8,9]. Additional “high-order” models were developed with through-the-thickness inserts to study localized bending effects from material discontinuities [2,10,11,12,13]. There are several papers that compare numerical analysis to experimental testing where failure modes of typical potted inserts are investigated [14,15,16,17,18,19,20,21]. Other studies focus on the crack initiation and propagation of partially potted inserts [22] and the influence of the position of the insert within the honeycomb core [23]. Other areas of sandwich composite joining methods focus on the load capacity of joints in shear and several other joining methods that do not use inserts [24].

Typical metallic inserts provide good strength and stiffness when resisting localized external loads. However, stress concentrations due to an abrupt change in stiffness, caused by both material and geometric discontinuities, can cause the joint to fail prematurely. There are several studies that introduce novel insert components; these include studies on revolute inserts in foam cores, non-standard metallic inserts in foam cores, combined additively manufactured core and insert designs, composite inserts, and scarf inserts [1,25,26,27,28,29,30,31,32]. Insert designs that used 3D manufacturing methods are explored in various studies. One study uses topology optimization and selective laser melting to develop an integrated aluminum insert that was bonded in place during the cure cycle [33]. Other 3D printed insert designs with various supporting structures, such as lattice, are investigated and compared to standard inserts [34]. Other studies investigate 3D printed insert designs with partially printed cores [35,36,37]. The studies and inserts described above have distinct differences from the inserts presented in this study, mainly in the exclusion of internal sweep cuts in the insert design.

Along with the wide range of insert designs, there is a wide range of materials that have been used to fabricate inserts. These materials include metals, plastics, and numerous composite combinations, as detailed in the studies described above. Studies on ULTEM and 3D printing have concentrated on many facets, such as mechanical properties, structural attributes, and processing factors. Aguilar [38] and Gallagher [39] conducted research on the mechanical characteristics of 3D-printed ULTEM 9085. Gallagher’s study additionally examined its performance in orbital environments. Gebisa [40], Zaldivar [41], and Basik [42] examined the impact of 3D printing process parameters and print orientation on the tensile properties of the material, respectively. These studies were conducted using a fused deposition modeling (FDM) process. Hossain [43] presented comprehensive analyses of the applications and determinants impacting the caliber of 3D printing. Hossain specifically addressed the obstacles and opportunities in this field. Chuang [44] performed additive manufacturing of aircraft engine components utilizing ULTEM 9085 and experimental ULTEM 1000 and compared these components with injection-molded components. These findings collectively suggest that ULTEM can indeed be used to produce high-quality and strong parts, particularly when the material and process parameters are carefully controlled to achieve the high performance desired in joint design.

This study compares the performance of standard metallic inserts to additively manufactured inserts made from ULTEM 9085 using an FDM process under pull-through loading. The inserts introduced in this study minimize the influence of discontinuities within the region around the insert to achieve higher maximum loading, stiffness, and total energy absorption. The manufacturing process and improvement in the metrics stated above show that these inserts can replace standard metallic inserts during pull-through loading for sandwich panels consisting of aluminum honeycomb cores.

## 2. Experimental Analysis

The sandwich composite material system used in this study consists of carbon fiber reinforced facesheets with an aluminum honeycomb core. The carbon fiber/epoxy is a plain weave prepreg from TORAY T700SC-12K-50C/#2510 [45]. All specimens had a single ply for each facesheet. The honeycomb core is HexWeb^®^ CRIII 5052 from Hexcel (Stamford, CT, USA) [46]. The density and thickness of the core used for each specimen is 97.71 kg/m^3^ and 12.7 mm, respectively. The potting material used for each specimen is a two-part epoxy from 3M (Saint Paul, MN, USA) which is SCOTCH-WELD™ DP420NS that was applied with an applicator and nozzle.

Two types of inserts were tested to benchmark the additively manufactured insert against the baseline. The baseline panel configurations used standard aluminum inserts with part number NAS1834A6-500 distributed by Clarendon Specialty Fasteners (Long Beach, CA, USA) (referred to as “Standard Inserts”). These are fully potted inserts with a through hole to accommodate M8 bolts. The additively manufactured panel configurations used inserts that were fabricated using a thermoplastic filament from Stratasys with part number ULTEM 9085 Resin (referred to as “AM Inserts”). The inserts were manufactured using a Fortus 450mc 3D printer manufactured by Stratasys (Eden Prairie, MN, USA) using an FDM process.

**Figure 1:**
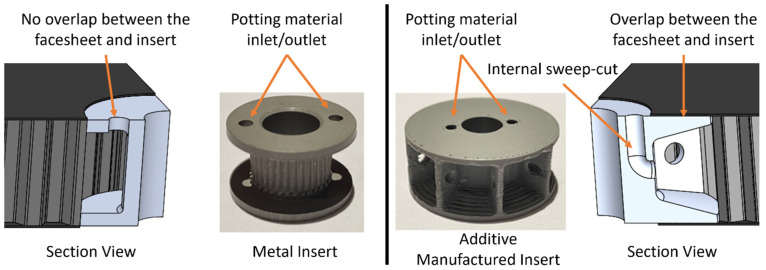
Inserts and CAD model section view of the Standard Insert (**left**) and the AM Insert (**right**).

The baseline test specimens using the standard inserts required a post-cure machining operation. A through-hole the same size as the insert diameter is drilled through the sandwich panel so the insert can be placed in the panel. After the insert is in place, it is set with the potting material described above and as directed by the manufacturer. There was no surface pretreatment of the sandwich panel or the insert before the potting material was applied. The AM inserts were integrated into the panel before curing and only required potting after the cure cycle, which eliminates the post-cure machining operation. Additive manufacturing makes it possible to include internal sweep cuts in the insert that allows potting material to flow around the insert and fill adjacent areas of the core. Since the AM inserts are integrated into the panel before curing, this also allows the top and bottom of the insert to overlap and adhere to the facesheets (shown in Figure 1). Due to the manufacturing method, standard inserts do not have any overlap from the facesheets and are only secured in place with potting material. Further, the diameters of the standard inserts and the AM inserts are 22 mm and 30 mm, respectively. This allows the potting material to disperse over a greater distance throughout the core in the panels using AM inserts. Additionally, additive manufacturing allows the stiffness of the insert to gradually increase, which reduces stress concentrations caused by abrupt changes in stiffness. Figure 1 shows the inserts and section views of the CAD models.

Each test specimen was cut from a 304.8 mm × 304.8 mm panel fabricated in a 210 min cure cycle. There are three phases of the cure cycle: Phase 1 includes a ramp up in temperature and pressure from ambient values to 132.2 °C and 170 kPa, respectively. This phase lasts for 60 min. Phase 2 lasts for 120 min and holds the temperature and pressure constant. Phase 3 is a 30 min ramp down back to ambient levels. Table 1 outlines the physical properties of each panel that was tested. Although the panels with AM inserts have larger diameters and the potting material was dispersed over a greater distance in the core, there is only a minor increase of 5.0% in the density of the overall panel. This is due to the density of the standard insert being 2.7 g/cm^3^ while the density of the AM insert is 1.27 g/cm^3^.

**Figure 2:**
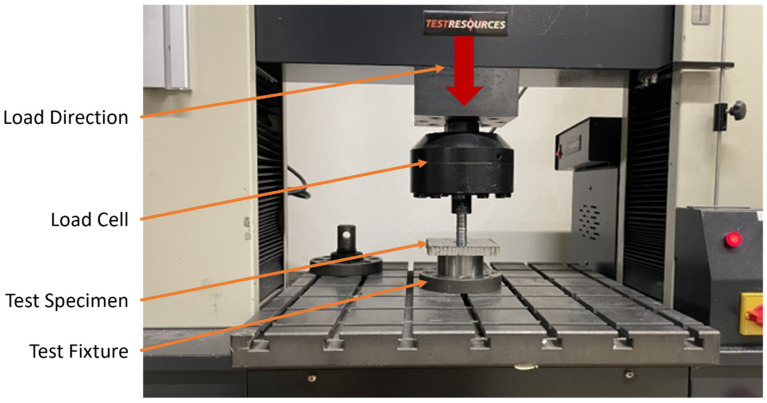
Pull-through test setup.

Pull-through tests were conducted using three specimens from each configuration and were carried out in accordance with the recommendations given by the manufacturer [47]. These recommendations include fixture specifications, specimen size, and reporting metrics. Testing was conducted using displacement control of 6.35 mm/min and was conducted at room temperature. Before each test was conducted, a torque wrench was used to apply 6.8 N-m of torque to each nut, clamping down on the insert. A schematic of the pull-through test and specimen is shown in Figure 2.

## 3. Numerical Analysis

Finite element models were created to simulate pull-through tests for both the standard and AM insert configurations to study the influence each insert has on the local stresses of the honeycomb core, debonding stresses between the facesheets and core, and overall deflections of the panel along each of their midplanes. Debonding stresses are defined as the maximum shear stress at the boundary between the core and the facesheet,

(1) $$\tau_{max}=\frac{\left(\sigma_{1}-\sigma_{3}\right)}{2}$$

where τ_max_ is the maximum shear stress, σ_1_ is the first principal stress, and σ_3_ is the third principal stress. The core is modeled explicitly using 2D elements due to the thin nature of the aluminum foils with isotropic linear elastic material properties. The facesheets are modeled using 3D hexagonal elements using orthotropic linear elastic material properties. The test fixture is modeled with 3D tetrahedral elements using an isotropic linear elastic model. It is assumed that the potting material fills up core cells that are exposed to areas where resin can freely flow. The potting material is modeled with 3D tetrahedral elements using an isotropic linear elastic material model. The standard insert and the AM insert are modeled using tetrahedral elements with an isotropic linear elastic material model and orthotropic linear elastic material model, respectively. Mechanical properties of each constituent are shown in Table 2.

**Figure 3:**
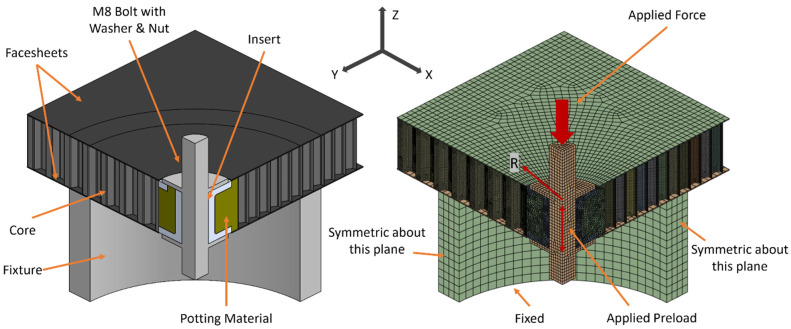
CAD model defining components (**left**) and FEM showing boundary conditions (**right**).

Contacting surfaces between the test fixture and the sandwich panel are modeled as frictional contact using a normal Lagrange formulation to enforce a near-zero displacement between contacting surfaces. Studies have shown that carbon fiber reinforced composites have a coefficient of friction between 0.15 and 0.35 depending on specific applications [48]. This study specifies a coefficient of friction of 0.15. A sensitivity study was conducted to check the influence the coefficient of friction has on the results of the model where the coefficient of friction was varied between 0.0 and 0.9 in 0.1 increments. The change in results among all simulations was less than 3%; verifying this parameter has minimal influence on the critical results. The simulation is non-linear due to (1) surface-to-surface contact between the test fixture and test specimen and (2) accounting for changes in geometric stiffness due to changes in shape (large deflection mode). The simulation consisted of two load steps: (1) apply bolt preload due to torquing the nut to 6.8 N-m and (2) apply 1600 N of external load. This approach allows stresses induced by bolt preload to be accounted for during the remainder of the pull-through test. The following equation was used to convert bolt torque to bolt preload:

(2) $$F_{i}=\frac{T}{kd}$$

where F_i_ is the bolt preload, T is the applied torque, d is the nominal bolt diameter, and k is a constant that depends on bolt material and size [49]. Mild steel bolts between 6 mm and 25 mm typically use a k value equal to 0.2 [49]. This results in a bolt preload value of 4250 N. The stresses in the inserts caused from this preload value are below the yield strength of the aluminum insert and compressive strength of the ULTEM 9085 insert, respectively. This ensures material non-linearity does not have to be accounted for within the bounds of this numerical investigation. A double-symmetric model was created to reduce computational requirements. All boundary conditions are shown in Figure 3 with corresponding mesh statistics shown in Table 3. Numerical simulations were conducted using Ansys Workbench 2023R2 (version 23.2.0.0) [50].

**Figure 4:**
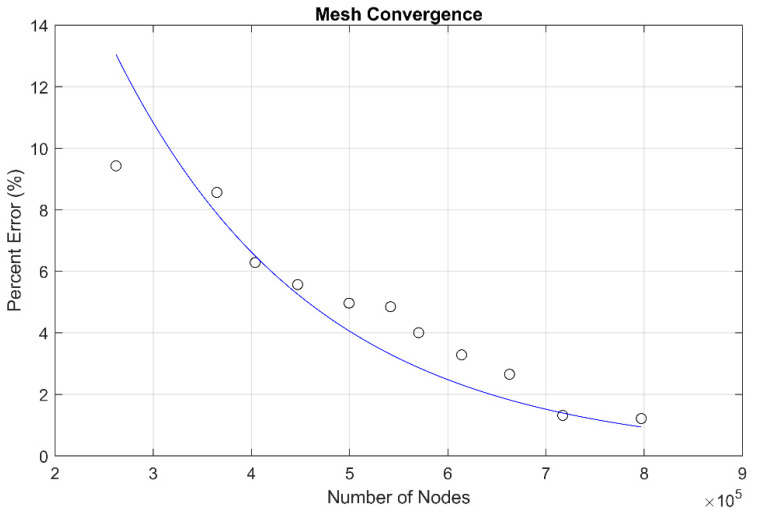
Mesh convergence results.

A mesh convergence study was conducted to determine the influence of mesh density on the results. Figure 4 shows the percent change in midplane deflections when compared to the model used for this study which has a final node count of 887,614. The data points show that when the model node count reaches 700,000, there is little change in results. Figure 4 is indicative of the other results that are displayed throughout this research. Additionally, the standard insert model and the AM insert model were meshed using the same metrics and the numerical results presented in this study are normalized and not presented as absolute. This approach further removes error caused by mesh convergence but still allows for the determination of percent changes in stress results between the different insert configurations.

## 4. Results and Discussion

Individual test values and average values for stiffness, maximum force, and total energy absorption are tabulated in Table 4. Total energy absorption is determined by calculating the area under the force vs. displacement curves for each specimen. The area up to the maximum load was considered for total energy absorption calculations. Since the AM inserts achieved higher maximum loads, they also achieved higher total energy absorption values. On average, when compared to the standard inserts, the AM inserts achieved 53.0% higher maximum forces, a 7.1% increase in stiffness, and a 62.3% increase in total energy absorption.

**Figure 5:**
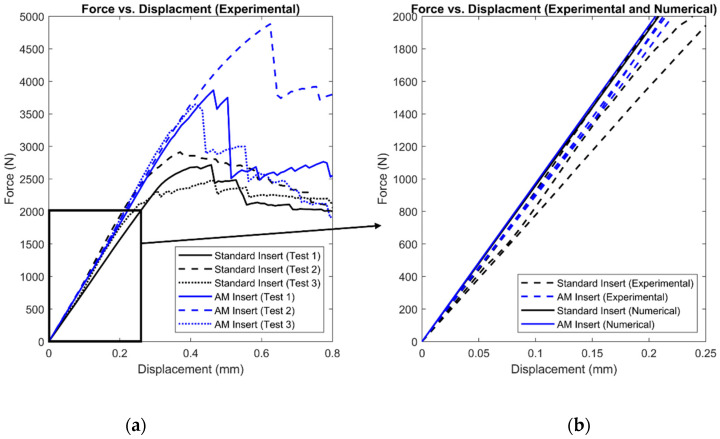
Force vs. displacement of experimental results (**a**) and a comparison of experimental and numerical force vs. displacement (**b**).

Force vs. displacement among the different panels are plotted in Figure 5 (left side of figure). The data show that the maximum force for the AM inserts was greater than the maximum force for the standard inserts. Along with the individual test results, the figure also shows a comparison of stiffness between the experimental results for the standard inserts and AM inserts and the numerical results (right side of figure). The average experimental stiffness of the standard inserts was 8641 N/mm while the stiffness in the numerical model was 9591 N/mm. This is a percent error of 9.9% between the experimental and numerical stiffnesses for the standard insert panels. The average experimental stiffness of the AM inserts was 9256 N/mm while the stiffness in the numerical model was 9722 N/mm. This is a percent error of 4.8% between the experimental and numerical stiffnesses for the AM insert panels. These percent errors demonstrate that the numerical models are simulating the experimentation accurately.

**Figure 6:**
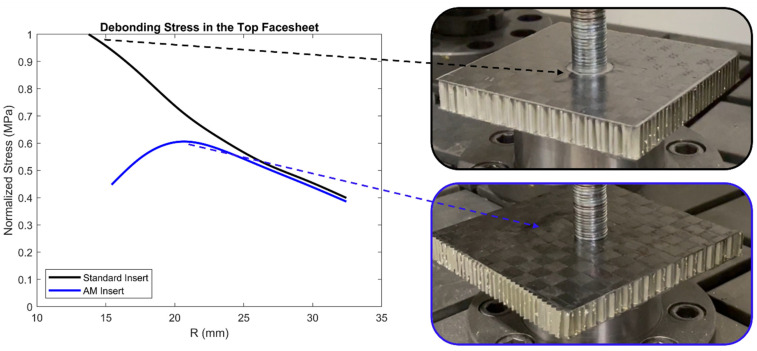
Finite element model debonding stress with corresponding initial failure location.

Experimental analysis shows that the first visible failure mode for both the standard insert panels and AM insert panels was the debonding of the top facesheet. Initial debonding occurs locally in the standard insert and AM insert when distance R (radius outward from the center of the insert) is 14 mm and 19 mm, respectively. This initial debonding distance correlates with the highest debonding stress seen in the FE models. This comparison with initial failure of the panels is shown in Figure 6 with the debonding stress normalized along the *y*-axis. Normalized debonding stress in the top facesheet shows there is a 38% reduction in maximum debonding stress in the AM insert when the same 1600 N external force is being applied. Similar initial debonding of the top facesheet occurred for all the panels that were tested.

**Figure 7:**
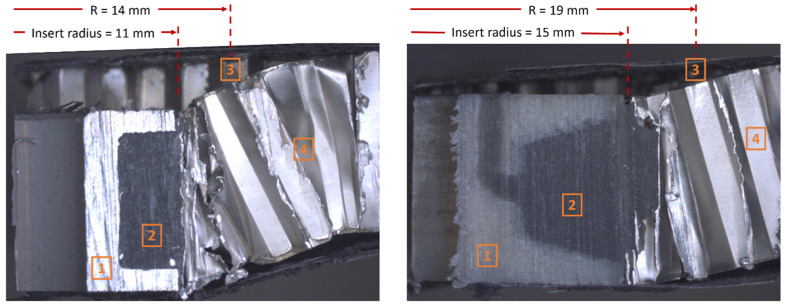
Section-cuts of the test panels with a standard insert (**left**) and AM insert (**right**).

The test panels were cut into sections to determine failure modes at final failure and to view the interaction between each of the constituents. Items 1 and 2 in Figure 7 are the insert and potting material, respectively. The potting material is fully bonded to the insert and the surrounding core cells. The image of the AM insert shows the effectiveness of the sweep cut obtained through the 3D printing process (FDM). Item 3 shows the initial debonding failure distance, as seen in Figure 6. Due to the larger diameter of the AM inserts, the dispersion of the potting material is pushed out at a greater distance R which corresponds to the initial debonding failure being pushed out. Although initial debonding occurs at different R distances, the distance between the initial debonding location and the edge of the insert is similar for both inserts and is equal to 3 mm for the standard insert and 4 mm for the AM insert i.e., [initial debonding distance, R]—[insert radius]. The reduced debonding stresses in the AM inserts can be attributed to the larger bonding area of the potting material to the facesheets facilitated by the manufacturing process. Item 4 shows the extent of core shear at final failure.

**Figure 8:**
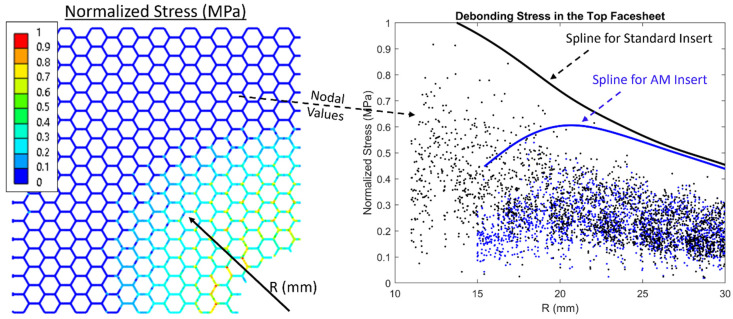
Splines capturing normalized debonding stress nodal values in the top facesheet for the standard insert and AM insert.

The finite element models correlate well with the experimental results capturing the stiffness (Figure 5) and the initial location of debonding in the top facesheet (Figure 6). These results provide confidence in comparing stresses internal to the sandwich panel which are hidden from view during testing. Nodal stress values were processed to determine the percent change in the quantity being considered. Normalized debonding stress in the top facesheet is compared to experimental results in Figure 6. Figure 8 shows an example of how splines were generated based on the data from the numerical models. First, nodal stresses were captured for the stress quantities being considered as a function of R. These values were then normalized to the highest value seen between the standard insert and the AM insert. From here, a cubic smoothing spline with a smoothing parameter of 0.25 was used to capture the 99th percentile of data for any given R distance. Figure 8 shows a reproduction of how the numerical results were captured for the standard insert and AM insert that was shown in Figure 6. 

**Figure 9:**
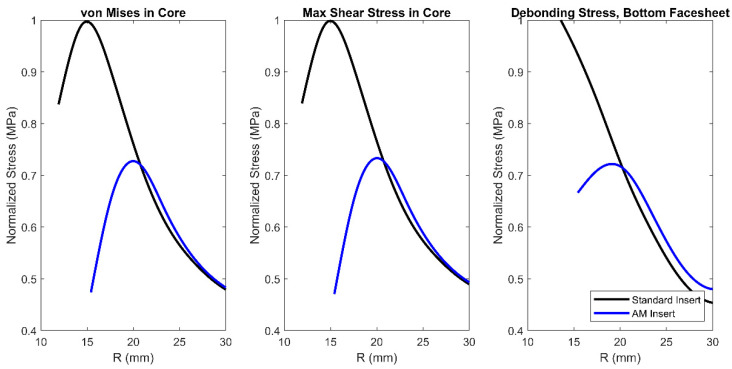
Normalized finite element model results for stresses in the core and debonding stress at the bottom facesheet.

Using the same methodology that was described above and shown in Figure 8, other stress quantities were evaluated. These stress quantities were von Mises stress in the core, maximum shear stress in the core, and debonding stress in the bottom facesheet. All these quantities were evaluated as a function of R. The trends are similar to the results shown for debonding stress in the top facesheet. There were reductions of 26%, 25%, and 27% for von Mises stress in the core, maximum shear stress in the core, and debonding stress in the bottom facesheet, respectively. These results are shown in Figure 9.

In addition to the comparisons shown above between the standard insert and the AM insert, a study was conducted to investigate the influence of taking the standard insert and changing only the material properties from aluminum to ULTEM 9085. When this was completed, there was a small change in debonding stresses in the top and bottom facesheets accompanied by no change in von Mises stresses and maximum shear stresses in the core. The reduction in debonding stresses in the top and bottom facesheets were 12% and 7%, respectively. Again, the reduction in debonding stresses in the top and bottom facesheet using the AM insert was 38% and 27%, respectively. This suggests that although there may be some benefit in simply changing materials from aluminum to ULTEM 9085, there is a much larger benefit to altering the geometry of the insert and taking advantage of the 3D printing process.

**Figure 10:**
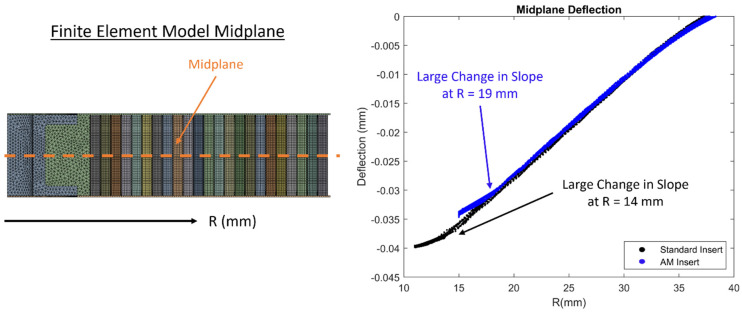
Midplane deflection at 400 N of applied force.

Figure 10 shows a comparison of deflections from each node along the midplane of the sandwich panels for both the standard insert and AM insert when the external force was 400 N. There is a substantial change in slope in the overall deflections in the sandwich panel for both insert configurations. For the standard insert panel, the substantial change in slope occurs when R is equal to 14 mm while for the AM insert panel, the substantial change in slope occurs when R is equal to 19 mm. These values have the same value for R as the stress metrics described above and shown in Figure 9. The AM insert design allows for potting material to disperse over a greater distance R which allows for the overall stiffness of the panel to increase more gradually as R approaches 0. The standard insert has a larger change in overall stiffness which is captured in Figure 10.

Results from both the experimental analysis and the numerical analysis show that the AM inserts perform better during pull-through testing for all metrics considered. Experimental analysis shows a 53.0% higher maximum force achieved and a 62.3% higher total energy absorption. These results are confirmed with numerical analysis showing stress levels are reduced for all quantities evaluated when the applied external force is the same. There was a 38% and 27% reduction in debonding stress in the top and bottom facesheet, respectively. There was also a 26% and 25% reduction in von Mises stress and maximum shear stress in the core, respectively. Additionally, when midplane deflections were probed, there was a substantial change in slope of the deflected shape for both insert configurations. The substantial change in slope in the overall deflections corresponds to the same locations where stresses are largest and where the first visible signs of failure occurred in the panels. These locations are R equal to 14 mm and R equal to 19 mm for the standard insert panels and the AM insert panels, respectively. The initial failure of the panels that was visible was the debonding of the top facesheet. However, stress results for the core shown in Figure 9 suggest that the initial failure could possibly have been core shear, or a combination of debonding and shear, which is internal to the panel and hidden from view.

## 5. Conclusions

A novel approach to the sandwich composite insert design was investigated using additive manufacturing technology. The material used for the additively manufactured insert was ULTEM 9085 Resin from Stratasys. The insert design presented here offers several advantages in both the fabrication process and overall performance when compared to a standard purchased aluminum insert. When fabricating the panels, the additively manufactured inserts are integrated into the panels prior to curing which eliminates a post-cure machining process. This type of integrated part allows the facesheets to overlap and adhere to the insert providing extra strength. The integrated design also allows for the overall insert diameter to be larger, allowing the potting material to disperse over a greater distance from the center of the insert through internal sweep cuts that are only achieved through the benefits of additive manufacturing. This allows the additively manufactured inserts to have a larger area of adhesion between the potting material and the facesheets when compared to the standard inserts. Although there is more potting material used, the density of the panels with additively manufactured inserts was only slightly more than the panels with standard inserts with a 5.0% increase. This is attributed to the density of ULTEM 9085 being less dense than aluminum.

Experimental evaluation demonstrated that the panels fabricated with additively manufactured inserts have an increase in stiffness, maximum force, and total energy absorption of 7.1%, 53.0%, and 62.3%, respectively. The increase in each of these metrics is beneficial and would allow components using this design approach to accommodate higher maximum loading. The first visible failure of all the panels tested was the debonding of the top facesheet and occurred at a distance of 14 mm and 19 mm from the center of the insert for the standard insert panels and the additively manufactured insert panels, respectively. The finite element models showed a reduction in von Mises stress in the core, maximum shear stress in the core, debonding stress in the top facesheet, and debonding stress in the bottom facesheet of 26%, 25%, 38%, and 27%, respectively. Although the initial failure of the panels that was visible was the debonding of the top facesheet, core stress results captured by the finite element models suggest that the initial failure could possibly have been core shear or simultaneous debonding and core shear. The results listed above for the experimental and numerical analyses indicate that the additively manufactured inserts performed better than the standard inserts during pull-through testing. Future studies should be aimed at optimizing insert geometry using internal sweep cuts and quantifying other test metrics, such as combined pull-through and shear loading.

## Figures and Tables

**Table 1:** Test panel physical properties.

Component		Mass (g)	Facesheet Thickness (mm)	Length (mm)	Width (mm)	Height (mm)	Volume (cm^3^)	Density (g/cm^3^)
Standard Insert	Panel 1	27.7	0.33	101.6	101.6	13.0	134.3	0.206
	Panel 2	27.5	0.34	101.9	100.8	13.1	134.9	0.204
	Panel 3	27.7	0.29	101.4	101.1	13.0	133.6	0.207
	Mean	27.6	0.31	101.6	101.2	13.1	134.3	0.206
AM Insert	Panel 1	31.7	0.32	109.1	104.2	13.1	148.6	0.213
	Panel 2	34.0	0.29	107.5	108.1	13.4	155.3	0.219
	Panel 3	31.8	0.23	107.1	104.8	13.2	147.6	0.215
	Mean	32.5	0.27	107.9	105.7	13.2	150.5	0.216
% Increase for AM Insert		-	-	-	-	-	-	5.0

**Table 2:** Mechanical properties of the core, facesheets, fixture, potting material, and inserts.

Material	E_x_ (GPa)	E_y_ (GPa)	E_z_ (GPa)	G_xy_ (GPa)	G_yz_ (GPa)	G_xz_ (GPa)	ν_xy_	ν_yz_	ν_xz_
Facesheet: CFRP	24.0	23.5	1.6	4.2	3.5	3.5	0.04	0.30	0.30
Core: Aluminum 5052	1.7	1.7	2.4	1.0	0.5	0.3	0.99	0.33	0.33
Fixture: Steel	200.0	200.0	200.0	76.9	76.9	76.9	0.30	0.30	0.30
Potting Material	3.8	3.8	3.8	1.4	1.4	1.4	0.35	0.35	0.35
Standard Insert: Aluminum	71.0	71.0	71.0	26.7	26.7	26.7	0.33	0.33	0.33
AM Insert: ULTEM 9085 Resin	2.4	2.4	2.4	0.9	0.9	0.9	0.30	0.30	0.30

**Table 3:** Finite element model statistics.

	Standard Insert					AM Insert				
	Facesheets	Core	Insert	Potting Material	Fixture	Facesheets	Core	Insert	Potting Material	Fixture
Element Type	Hex20	Quad4	Tet10	Tet10	Hex20	Hex20	Quad4	Tet10	Tet10	Hex20
Element Count	48,630	365,321	27,762	73,550	4092	51,158	352,715	72,026	117,649	2202
Node Count	343,826	374,977	42,539	108,321	17,951	361,602	362,206	107,969	173,289	10,667
Avg. Aspect Ratio	1.7	1.2	1.8	1.9	3.1	1.8	1.2	1.8	1.9	2.4

**Table 4:** Summary of test results.

Component	Stiffness (N/mm)			Mean	CV%	Maximum Force (N)			Mean	CV%	Energy Absorption (J)			Mean	CV%
	Test 1	Test 2	Test 3			Test 1	Test 2	Test 3			Test 1	Test 2	Test 3		
Standard Insert	7811	9860	8251	8641	10.2	2715	2912	2473	2700	6.6	0.759	0.618	0.768	0.715	9.6
AM Insert	8710	9107	9951	9256	5.6	3864	4881	3647	4131	13.0	0.955	1.710	0.816	1.160	33.9
% Increase for AM Insert	-	-	-	7.1	-	-	-	-	53.0	-	-	-	-	62.3	-

## Data Availability

The original contributions presented in the study are included in the article, further inquiries can be directed to the corresponding author/s.
